# A searchable cross-platform gene expression database reveals connections between drug treatments and disease

**DOI:** 10.1186/1471-2164-13-12

**Published:** 2012-01-10

**Authors:** Gareth Williams

**Affiliations:** 1 Wolfson Centre for Age-Related Diseases, King's College London, London Bridge, London SE1 1UL, UK

**Keywords:** Global gene expression, connectivity map, microarray, cancer, neurodegenerative disease, Alzheimer's disease

## Abstract

**Background:**

Transcriptional data covering multiple platforms and species is collected and processed into a searchable platform independent expression database (SPIED). SPIED consists of over 100,000 expression fold profiles defined independently of control/treatment assignment and mapped to non-redundant gene lists. The database is thus searchable with query profiles defined over genes alone. The motivation behind SPIED is that transcriptional profiles can be quantitatively compared and ranked and thus serve as effective surrogates for comparing the underlying biological states across multiple experiments.

**Results:**

Drug perturbation, cancer and neurodegenerative disease derived transcriptional profiles are shown to be effective descriptors of the underlying biology as they return related drugs and pathologies from SPIED. In the case of Alzheimer's disease there is high transcriptional overlap with other neurodegenerative conditions and rodent models of neurodegeneration and nerve injury. Combining the query signature with correlating profiles allows for the definition of a tight neurodegeneration signature that successfully highlights many neuroprotective drugs in the Broad connectivity map.

**Conclusions:**

Quantitative querying of expression data from across the totality of deposited experiments is an effective way of discovering connections between different biological systems and in particular that between drug action and biological disease state. Examples in cancer and neurodegenerative conditions validate the utility of SPIED.

## Background

There is a wealth of deposited gene expression data available for downloading and/or online interrogation. For example, the NCBI gene expression omnibus (GEO) [1] hosts over half a million single array chip expression profiles and the EBI hosts the ArrayExpress [2] database with a similar largely overlapping number of arrays. These data cannot be compared directly as they come from different array platforms covering many different species and a variety of normalisation schemes are used. In the overwhelming number of analyses expression profiles are compared within the given series and probed for the up or down regulation of single genes using volcano plot representations or other statistical filters [3]. Alternatively, a larger set of responders can be scored against gene sets corresponding to pathways [4], interacting networks [5-7] or gene ontology classes [8,9]. For large series it is possible to compile correlations of expression changes of individual gene pairs and groups of genes leading to a hierarchical clustering based network discovery and gene interaction prediction. To this end SOURCE [10] hosts gene expression profiles across a large collection of experimental series and profile correlations within a given series can be examined to predict genes with similar or related function. Many array analysis applications incorporate array derived network data that are valuable aids in characterising the expression profile data (Ingenuity Pathways Analysis (IPA) (Ingenuity^® ^Systems, http://www.ingenuity.com); GeneGo (GeneGo, Inc., St. Joseph, MI)). However, these analyses do not allow for a direct quantitative comparison between separate expression studies and therefore a lot of the information contained in the experiment is effectively lost.

The idea that transcriptional change profiles can be directly compared to asses drug target specificity was demonstrated in yeast systems by Marton *et al *[11] and later extended by Hughes *et al *[12]. The connectivity map (CMAP) project sought to apply these ideas to generate a database of drug perturbagen transcriptional profiles that can be searched with transcriptional responder sets by third parties to match phenotype to drug treatment [13]. In this methodology the expression change profile as a whole defines the biological perturbation and not a relatively small selection of down- or up-regulated genes. An important point here is that biological effects are not necessarily caused by the corresponding transcriptional changes. Rather, the underlying assumption is that correlations in transcriptional change profiles are reflected in similar biological responses. One powerful application of the CMAP is the matching of disease state to drug treatment. In simple terms, if a disease state is reflected in a well defined transcriptional response, then a drug that has the opposite effect on expression of these transcripts might be of therapeutic value. The fundamental assumption here is that there is a degree of overlap in the transcriptional changes induced by the same perturbagen in different cell contexts. In particular the CMAP consist of expression change data for human cancer cell lines and it is hoped that there is a degree of universality that will enable useful predictions to be made as to the action of the drugs in different cell types. Of course, the successful application of the CMAP should encourage rather than hinder the inclusion of other cell types more relevant to the type of biological system under investigation. At the present the CMAP consists of expression change fold profiles for 6,100 single treatments versus control pairs for a collection of 1,309 drug like perturbagens. Results are collected from treatments of four distinct types of human cancer cell lines. The CMAP database can be interrogated with expression change signatures consisting of lists of up and down regulated probe sets. Correlation both in the positive and negative sense are scored by means of a non-parametric Kolmogorov-Smirnov (KS) statistic [14]. The remarkable observation was that signatures from published studies showed correlation with CMAP profiles for drugs known to act against the same targets. This has opened the way for the CMAP to be used as a drug discovery tool where it is probed with signatures encoding disease states.

If the CMAP methodology is accepted as a useful discovery tool then it is natural to look for ways of extending it to incorporate expression data from a wider set of experiments. There are obvious advantages to having this kind of database, for example it will open up a large number of different samples and treatment conditions for direct interrogation. This was the idea behind GEM-TREND [15], where 26,000 expression samples from various platforms and species were compiled into a searchable database. The search methodology mirrors that of CMAP in that the database consists of ranked lists of genes and it is interrogated with up and down regulated gene sets and query signatures are scored by a KS statistic with the significance based on reference to random gene set scores. One difference to the CMAP database is necessitated by the multiple origins of the expression profile data represented by multiple probe ID definitions. The problem of multiple probe IDs is solved by the GEM-TREND database having expression profiles mapped onto UniGene IDs. The database consists of experimental series where samples can be clearly assigned to treatment and control groups. Of course, this is not always the case and this limits the scope of the database.

In compiling the expression database SPIED we sought to loosen the restraints inherent in previous treatments and thereby open up a larger set of data for interrogation. In many expression series sets there is no clear control/treatment assignment or there could be multiple alternative reference profile definitions. To address this problem of generating fold change profiles without reference to a defined control, an effective fold (EF) has been introduced corresponding to the expression level relative to the experimental series average. In this way, data can be compiled automatically without the need for manual inspection. In cases where the experimental series consists of well defined multiple treatment and control samples the fold profiles are usually given by the ratio of the average treatment to average control values. In general this fold profile will have high positive correlation with the EF profiles from the treatment set and high negative correlations with the control set. In cases where there is no obvious way of separating samples into control and treatment sets, as with samples from multiple organ types or cell types, the EF representation can be viewed as a normalized expression value. In searching SPIED with a query profile one is not deriving any biological significance for non-correlating profiles as lack of correlation can be attributed to multiple factors such as bad experimental data or genuine lack of biological relevance. Rather significantly correlating or anti-correlating profiles are posited as having biological significance. The next objective was to reduce the expression profiles to non-redundant EF gene profiles by associating each gene with just one probe ID, so that the database can then be searched with gene set data alone. Here, for a given chip platform the distribution of each probe ID EF value across the totality of series was compiled and each gene was then assigned to the probe having the highest average fold magnitude. The gene names were unambiguously associated with the Entrez human gene list (http://www.ncbi.nlm.nih.gov), consisting of 24,764 genes and these were matched to probe IDs by inspection of the given platform annotation files. The final form of SPIED consists of individual files for each chip platform and these files are formatted starting with a gene list followed by the sample ID and corresponding EF profiles. This format lends itself to rapid searching in an analogous fashion to FASTA formatted sequence databases. In contrast to the KS query score scheme, which requires generating random reference gene list data, we adopted a simple regression scoring scheme with corresponding statistic. Searches can be performed on a standard desktop PC and take ~10 minutes per query. Although, the present database consisting of expression data for over 100,000 samples from five platforms covering three species (human, mouse and rat) is all from Affymetrix expression array chips, the methodology is truly platform independent and it is a straight forward matter to include data based on other array technologies. Other species and platform technologies will be added to SPIED in the future. For the present study Affymetrix was chosen because of the relatively large number of available samples. Further details are presented in the methods section below.

## Results

### Drug treatment based profile SPIED queries

The CMAP contains expression change profiles as ranked array probe IDs for 6,100 individual treatments corresponding to 1,309 distinct drug-like compounds. Statistically filtered response profiles can be defined for 1,218 of the drugs as these have at least three instances in the database. The profiles can be mapped onto a non-redundant gene list by uniquely associating one probe ID to a given gene and dropping the other probe ID for this gene with less robust expression changes over the database. This is the same methodology underlying the SPIED database. We took the responder profiles for the 1,218 drugs and searched the SPIED for maximally (anti-)correlated expression change profiles. The objective is to see to what extent the CMAP transcriptional signatures correlate with transcriptional responses assimilated within our platform independent database of over 100,000 microarrays deposited by a very large number of groups to the public domain.

**Figure 1:**
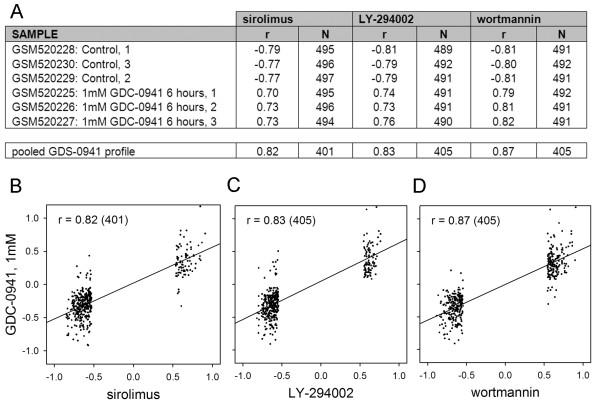
**High scoring correlations in the SPIED with queries derived from the CMAP profiles of rapamycin, LY-294002 and wortmannin are with a pI3 kinase inhibitor GDS-0941**. The individual sample scores and the regression scores (in this and later Figures/Tables *r *is the Pearson correlation coefficient and *N *is the number of genes in the given correlation) with the treatment and control groups are shown in A. The pooled profiles are defined by the ratios of the treatment to control averages. The fold regression plots are given in B, C and D.

**Figure 2:**
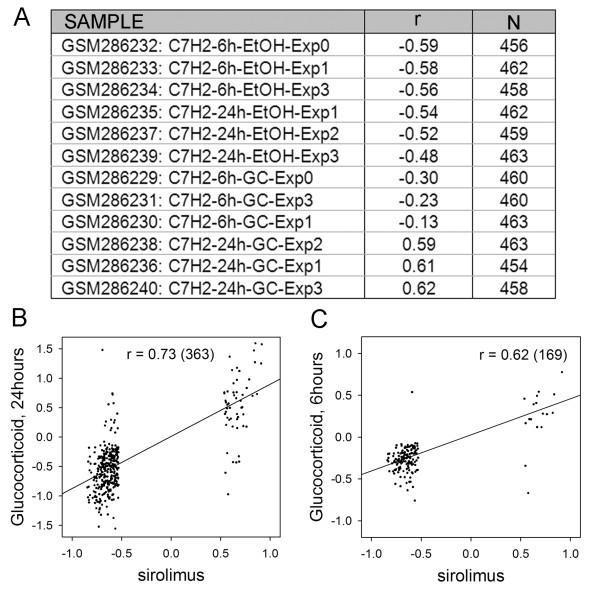
**The CMAP rapamycin query score highly against a glucocorticoid treatment experiment**. The correlations with the individual samples are shown in A and the regression plots for the pooled treatment and control groups are shown at 24 hours in B and at 6 hours in C.

**Figure 3:**
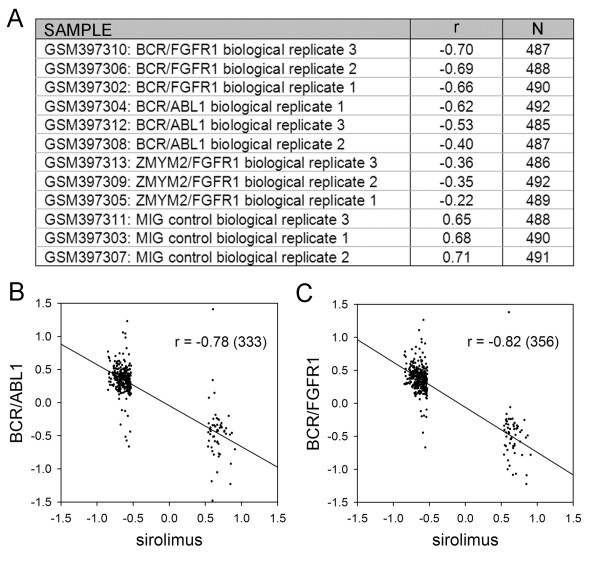
**The rapamycin CMAP significantly anti-correlates with the transcriptional changes induced in chord blood cells by two distinct BCR fusion transformations**. The correlations with the individual samples is shown in A and the negative regression with the pooled BCR/ABL1 transform v control and BCR/FGFR1 transform v control are shown in B and C respectively.

The CMAP is well populated with drugs that target the same or different steps in the PI3K-mTOR signalling cascade. In this context the results for LY-294002 (PI3K inhibitor) (61 instances), rapamycin (mTOR antagonist) (44 instances) and wortmannin (PI3K/mTOR antagonist) (18 instances) showed a high degree of overlap (rapamycin v LY-294002 *r *= 0.90 *N *= 3565, rapamycin v wotmannin *r *= 0.91 *N *= 1849, LY-294002 v wotmannin *r *= 0.88 *N *= 2217, where *r *is the Pearson correlation coefficient and *N *is the number of shared genes with significant fold values), see additional file 1 for the full fold change data. It is a straightforward matter to query the SPIED with these drug expression profiles. This is done by calculating the regression scores against the individual SPIED entries and retaining the top ~100 correlations, see Methods for details. For simplicity and uniformity of treatment, unless otherwise stated, we query SPIED with expression profiles containing 500 genes with the largest fold values passing the p < 0.05 significance threshold. It should be noted that results will be largely insensitive to the size of the query profile. The top SPIED correlate for all three drugs was the Pan-PI3K inhibitor GDC-0941 treated T47D breast cancer cells and the regression scores for the tree query signatures against all 6 samples in the series are shown in Figure 1A. The high degree of correlation is illustrated by regression plots for the three query profiles against the pooled GDC-0941 profile, see Figure 1B, C, D. All three inhibitor queries also pick out mTOR antagonist studies [16], but a more interesting correlation is with a glucocorticoid (dexamethasone) treatment of acute lymphoblastic leukaemia (ALL) cells [17], the rapamycin scores are shown in Figure 2A. The correlation increases with the length of drug treatment, being higher at 24 hours, Figure 2B, C. This result reveals another connection between mTOR antagonism and the corticosteroid mechanism as it has been shown that corticosteroid resistance in ALL can be overcome by mTOR antagonism [18]. Chronic myeloid Leukaemia (CML) and some instances of ALL are the result of the ABL tyrosine kinase translocation and fusion to BCR, the BCR-ABL fusion event [19]. This pathology has been targeted with rapamycin and our results support this approach based on the high degree of anti-correlation of the CMAP rapamycin profile with a transcriptional profile of BCR fusion construct transformed chord blood cells. The correlation scores are shown in Figure 3A. There is a clear anti-correlation of rapamycin profile with the BCR-ABL profiles pointing to a possible reversal of the phenotype, Figure 3B. Also, there is a high anti-correlation with the BCR-FGFR1 profile indicating a possible therapeutic role of rapamycin, Figure 3C.

In the original CMAP presentation [13] it was shown that meaningful results can be obtained from anti-correlating profiles. In particular the estrogen transcriptional response was shown to anti-correlate with the profiles of estrogen antagonists fulvestrant, tamoxifen and raloxifene. In this context it is of interest to note that high scoring SPIED hits for all three antagonists corresponded to anti-correlations with estrogen treatment samples. We have shown one example in Table 1 corresponding to a estrogen, tamoxifen and an extract from the cimicifuga plant [20].

**Table 1:** Estrogen antagonist queries derived from CMAP corresponding to raloxfene, fulvestrant and tamoxifen significantly anti-correlate with an estradiol treatment experiment.

	raloxifene		fulvestrant		tamoxifen	
**SAMPLE**	**R**	**N**	**r**	**N**	**R**	**N**

GSM155008: 24h_Estradiol_1	-0.52	495	-0.58	496	-0.45	497
GSM155009: 24h_Estradiol_2	-0.52	496	-0.60	498	-0.43	498
GSM155007: 24h_DMSO_2	-0.21	498	-0.08	500	-0.26	499
GSM155006: 24h_DMSO_1	-0.10	493	0.13	492	-0.23	497
GSM155013: 24h_Tamoxifen_2	-0.01	500	-0.06	497	0.04	498
GSM155012: 24h_Tamoxifen_1	0.39	499	0.30	497	0.27	499
GSM155005: 24h_Cimicifuga_2	0.34	497	0.39	498	0.34	500
GSM154972: 24h_Cimicifuga_1	0.48	499	0.41	499	0.39	499
						
**Pooled profiles**						

Estradiol v DMSO	-0.63	58	-0.76	90	-0.46	47
Tamoxifen v DMSO	0.65	26	0.19	24	0.67	22
Cimicifuga v DMSO	0.58	70	0.33	75	0.56	66

Positive correlation is seen with tamoxifen treatment and a herbal extract cimicifuga. The regression scores for the pooled treatment and control groups are shown at the bottom of the table.

For illustration purposes we have shown the common high correlating hits for three separate histone deacetylase (HDAC) inhibitor profiles in the CMAP series. These are vorinistat, trichostatin A and valporic acid. In Table 2 we have shown the regression scores for the multiple HDAC inhibitor study with a colorectal carcinoma cell line [21]. The query results for all the above searches are given in additional file 2.

**Table 2:** The HDAC inhibitor queries trichostatin A, vorinostat and valproic acid derived from the CMAP highly correlate with an HDAC inhibition experiment.

	trichostatin A		vorinostat		valproic acid	
**SAMPLE**	**R**	**N**	**r**	**N**	**r**	**N**

GSM548552: 8146_vehicle control_2	-0.86	492	-0.89	494	-0.65	492
GSM548551: 8145_vehicle control_1	-0.85	490	-0.88	494	-0.62	491
GSM548546: 8140_LZ_2	0.65	491	0.62	492	0.50	485
GSM548545: 8139_LZ_1	0.66	491	0.61	489	0.52	485
GSM548547: 8141_SAHA_1	0.68	491	0.66	488	0.50	487
GSM548548: 8142_SAHA_2	0.70	492	0.70	489	0.51	485
GSM548550: 8144_FK228_2	0.72	493	0.79	488	0.40	484
GSM548549: 8143_FK228_1	0.75	492	0.81	484	0.45	484
						
**pooled profiles**						

LZ v control	0.89	388	0.91	382	0.74	303
SAHA v control	0.90	374	0.92	368	0.74	290
FK228 v control	0.88	387	0.92	390	0.69	301

The regression scores for the pooled treatment and control groups are shown at the bottom of the table.

Next we consider profiles derived from disease states. For brevity we focus on two unrelated pathologies: cancer and neurodegeneration.

### Querying SPIED with cancer derived profiles

**Figure 4:**
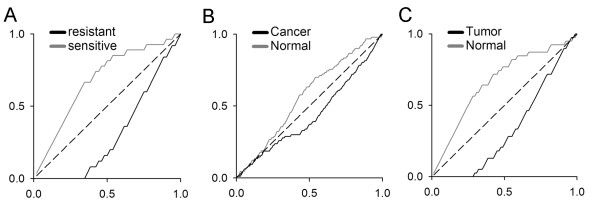
**Enrichment plots for high scoring SPIED hits against the glucocorticoid (dexamethasone) resistant v sensitive profile query**. The dexamethasone resistance query scores highly against a corticosteroid (prednisolone) resistance study. The enrichment plot is shown in A. Ordering the samples according to correlation score with the query profile the enrichment plot is the cumulative fraction of the given phenotype in the given sample fraction. Explicitly, if there are *N *correlation score ordered samples and two phenotypes defined by *q_i _*= ± 1 with *i *= 1,...,*N*, then the enrichment for the phenotypes is $e_{k}^{+/)-}=\pm \frac{\overset{k}{\underset{i=1}{\sum }}q_{i}}{\overset{N}{\underset{i=1}{\sum }}q_{i}}$and the enrichment plot is just $e_{k}^{+/)-}$against $\frac{k}{N}$. The plot shows that sensitive samples are enriched for lower correlation scores and resistant samples are enriched for higher correlation scores. The enrichment plots for the lung cancer study is shown in B and for the pancreatic cancer study in C.

The class of diseases with the most extensive repository of expression data is cancer and therefore a cancer disease profile search of SPIED will be an ideal testing ground for the methodology. The original CMAP disease application implicated mTOR inhibition as a target for imparting sensitivity to dexamethasone treatment resistant ALL [18]. We searched the SPIED database with the dexamethasone resistant v sensitive profile to see if there are common features in published transcriptional studies. The query profile consisted of the 500 most highly regulated genes that passed the lowest significance test of *p *< 0.05, see additional file 1. As with the SPIED profiles the query profile also consists of a non-redundant gene list. Not surprisingly, the highest correlation scores came from the experiments from which the query profile was generated, see additional file 2 file. In addition, we found a high correlation to an independent later study of ALL sensitivity to corticosteroid (prednisolone) treatment [22]. This study generated transcriptional profiles of ALL patient leukaemia cells with the objective of uncovering a gene signature that can predict the sensitivity to prednisolone treatment. Combining the 27 infant and non-infant corticosteroid sensitive samples and the 25 resistant samples we can define a statistically filtered sensitivity profile to make a direct comparison with the query profile and we find a high degree of correlation (*r *= 0.94), see additional file 2. When the high scoring sample belongs to a relatively large sample series and the phenotype is binary we can perform a non-parametric significance test to measure the extent of enrichment of the given phenotype for high or low correlation scores. For example in the last case there were 25 resistant and 27 sensitive samples. Ranking the samples according to their correlation with the resistant versus sensitive query profile we find 20 resistant samples in the top 25 and 22 sensitive samples in the bottom 27. This is highly significant and can be quantified with a simple Fisher exact test. Explicitly, the probability *p *of 20 or more resistant samples in the top 25 correlations is less than 9 × 10^-7^. The K-S significance score can be calculated by counting the number of times a random rearrangement of the samples gives a better enrichment, we find *p *< 3 × 10^-6^. The enrichment plot is given in Figure 4A. As expected the top scoring correlations were dominated by samples from blood derived cells, for simplicity we restricted our analysis to the top 100 most significantly correlating samples. However, two studies in unrelated tissue pathologies were highly correlated with the corticosteroid resistant profile. These were a comparison of lung epithelia with cancer in smokers [23] and a differential expression between healthy and cancerous pancreatic tissue [24]. The smoking study consisted of non-diseased lung epithelia from 187 individual smokers 97 of whom were diagnosed with lung cancer. Ranking the samples according to query correlation score we find that in the top 97 there are 64 cancer cases and in the bottom 90 there are 57 non-cancer cases, with a significance score of *p *< 5 × 10^-5^. The K-S significance is *p *< 2 × 10^-4^. The enrichment for positive correlations with the corticosteroid resistance profile in the cancer cases is shown in Figure 4B. Interestingly, it has been shown that there is a down regulation of the glucocorticoid receptor (GR) in small cell lung cancer [25,26] and reversing this promotes cancer cell apoptosis [27]. The pancreatic cancer study sought to establish a transcriptional signature of tumour versus normal pancreatic tissue by laser capture of cancerous and normal tissue from the same pancreas. In total 39 sample pairs were published and we find a high positive correlation with the corticosteroid resistance profile, *p *< 2 × 10^-6 ^and a K-S significance score of *p *< 3 × 10^-7^. The enrichment curve is shown in Figure 4C. In this context it has been reported that loss of GR expression has been seen in pancreatic carcinoma relative to normal tissue [28] and elevating GR expression has been shown to inhibit pancreatic tumour growth in a hamster model [29]. The query results are given in the additional file 2.

### Neurodegenerative Disease

**Figure 5:**
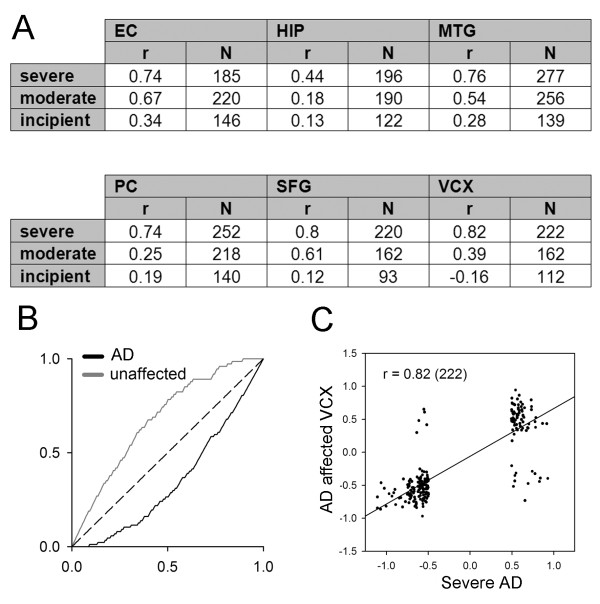
**A severe AD query is highly correlated with another AD transcription profiling study in SPIED**. The regression scores across six brain regions are shown in A. All apart from HIP are highly correlated with the severe AD profile. The overall enrichment for positive query correlation is shown in B (see Figure 4 legend for definition of enrichment plot) and a particular brain region regression plot is illustrated in C.

The analysis of gene expression changes associated with neurodegenerative disease has been hampered by the difficulty of extracting high quality RNA from post-mortem tissue [30,31]. One way of validating a disease associated gene expression profile is to show that it shares significant features with profiles derived from independent experiments on related pathologies. A positive result would validate the query profile and furthermore lead to a more robust core response profile based on multiple experiments. To this end we constructed three separate query profiles based on transcriptional profiles from the brains of patients with three degrees of severity of Alzheimer's disease (AD) [32], see additional file 1. The number of significant changes increases with severity of disease and we queried the SPIED with these three profiles, see additional file 2. Not surprisingly, the high scoring correlations are those from which the query profiles were derived. In addition to these the query returned correlations with other AD studies and various neurodegenerative diseases. The high scoring AD expression series was an extensive study of 161 samples from various brain regions of AD patients and age-matched controls [33]. Ignoring brain regions for now, there are 87 AD samples and 74 controls. Ranking the samples according to correlation score against the severe AD query profile we find a very significant enrichment of positive correlations with AD samples (*p *< 10^-8^, based on the Fisher test as above). Pooling the samples from the different brain regions results in significant correlations for 5 out of the 6 brain regions, see Figure 5.

In addition to AD correlations we found high scoring correlations with samples derived from Huntington's disease (HD), Down's syndrome (DS), Parkinson's disease (PD) and bipolar disorder (BD) brains. In this sense the profile cannot be considered to distinguish AD pathology from other degenerative diseases. However, it is of interest to examine in greater detail these cross-disease similarities. In particular, the severe AD query had a high correlation with severe stage HD caudate nucleus (CN) samples. The HD study consisted of 404 samples split across two platforms (201 samples on GPL96 and 203 on GPL97) in three brain regions from control and HD individuals [34]. The high correlation was with the GPL96 series. In terms of a binary Fisher analysis where brain region specificity is ignored, we get a small enrichment of *p *< 6 × 10^-3^. However, when the different brain regions are considered separately, we get significant regression scores in each region. The results are tabulated in Table 3.

**Table 3:** The severe AD profile query correlates with other neurodegenerative condition transcriptional changes.

Huntington's disease in various brain regions						
	**Caudate nucleus**		**Cerebellum**		**Frontal cortex**	

	**r**	**N**	**r**	**N**	**r**	**N**

**severe**	0.85	315	0.72	113	0.78	101
**moderate**	0.73	290	0.39	92	0.22	66
**incipient**	0.33	124	0.39	48	0.17	44
						
**PD in various brain regions**						

	**SFG**		**LSN**		**MSN**	

	**r**	**N**	**r**	**N**	**r**	**N**

**severe**	0.88	85	0.77	127	0.82	154
**moderate**	0.61	74	0.70	104	0.55	134
**incipient**	0.43	34	0.21	46	0.12	52
						
**Down's Syndrome**				**Bipolar Disorder**		
		
	**r**	**N**			**r**	**N**
		
**severe**	0.58	161		**severe**	0.87	139
**moderate**	0.68	202		**moderate**	0.58	73
**incipient**	0.04	112		**incipient**	0.03	44

The correlation scores with the severe AD profile are shown for four distinct neurodegenerative conditions: Huntington's disease, Parkinson's disease, Down's syndrome and Bipolar disorder.

The PD correlation was with a study of 94 samples from three different regions of diseased and normal brains [35]. Pooling samples according to brain region we find that the severe AD profile had a high correlation with all three regions studied: superior frontal gyrus (SFG) *r *= 0.88; lateral substatia nigra (LSN) *r *= 0.77; medial substantia nigra (MSN) *r *= 0.82, see Table 3.

The chromosome 21 trisomy underlying DS leads to the development of many of the characteristics of AD pathology [36,37]. Therefore, it is not surprising to find a high correlation in SPIED form a transcriptional profiling of DS brains. This study comprised 8 healthy and 7 DS individual brains [38]. Combining the expression data into a thresholded fold change profile we find that there is a significant but small positive correlation with the severe AD profile, with *r *= 0.58. Interestingly, the correlation is higher with the moderate AD profile, with *r *= 0.68, see Table 3.

The first transcriptional profiling of BD brains pointed to the down regulation of synaptic and mitochondrial proteins in the orbital frontal cortex [39]. This synaptic pathology picture of BD is further strengthened by our analysis of the AD profile correlates within SPIED. Pooling the 10 BD samples and 11 controls we find a high regression score with the severe AD profile, see Table 3[39]. It is important to note that this correlation is with a subset of the BD signature as it consists of genes that are also altered in AD. However, it is outside the scope of the present paper to combine profiles into disease specific queries.

Not surprisingly the high correlations are dominated by experiments on human samples. Perhaps of greater interest to the biologist are animal models of neurodegeneration. There has indeed been a debate as to the relevance of animal models of neurodegeneration to drug discovery, as age-related neurodegenerative conditions are rare in nonhumans [40]. In this context it is interesting to look at what correlations the AD query profile returns when we restrict the search to rodent platforms. The SPIED database contains samples from two murine and one rat platforms. Within the top 100 high scoring samples we have four separate studies directly relevant to neuropathology, see additional file 2. In particular, we find high scores with two separate spinal contusion models. The mouse experiments generated a post injury expression time series (GEO accession GSE5296, also http://pepr.cnmcresearch.org) and the AD profile correlation emerges at 72 hours post injury, see Table 4. The other spinal chord contusion study was in rats at 35 days post injury [41], see Table 4. In addition to these contusion models high scores were for a murine SOD1(G93A) mutant model of Amyotrophic lateral sclerosis (ALS) (GEO accession GSE18597) and a murine model of prion disease (GEO accession GSE23182). In the SOD1 (G93A) transcriptional profile series we found the correlation with AD emerging with older mice, with negligible correlation at the 28-70 day window and significant correlation with the 98-126 day late stage window profiles. This is consistent with the timescale of disease onset in the mouse model [42,43]. Prion disease is modelled in mice through ME7 prion agent infection resulting in both a behavioural phenotype and synaptopathy [44]. The transcriptional study corresponded to hippocampal profiles for ME7 v normal brain homogenate inoculated mice (GEO accession GSE23182). Pooling the treatment sets we get a good correlation with the AD profile, see Table 4. Thus it is clear that there is a core response profile shared across many neurodegenerative conditions and animal models of these conditions. Importantly, this core set is characterised by synaptic pathology and mitochondrial dysfunction, both of which are hypothesised to be causative of a number of neurodegenerative disease states.

**Table 4:** Rodent correlations with the severe AD profile.

	Mouse spinal chord injury v sham at injury site									
	
	4 h		24 h		72 h		7 d		28 d	
	
	R	N	r	N	r	N	r	N	r	N
**severe**	0.12	55	0.26	131	0.60	148	0.55	148	0.62	139
**moderate**	-0.08	66	0.12	138	0.45	162	0.47	164	0.52	129
**incipient**	-0.07	37	-0.08	62	0.31	92	0.25	82	0.34	77
										
										
	**Rat chronic spinal** **chord contusion**			**Prion infection** **neurodegeneration**			**SOD1(G93A)**			
							
							**young 28-40d**		**old 112-126d**	
					
	**r**	**N**		**r**	**N**		**r**	**N**	**r**	**N**
				
**severe**	0.76	76		0.70	176		0.07	109	0.49	205
**moderate**	0.63	63		0.66	158		-0.01	114	0.42	210
**incipient**	0.50	26		0.11	71		-0.08	76	0.19	105

The severe AD profile is significantly correlated with four different rodent models of neurodegeneration and nerve injury. The correlation in the spinal chord injury time course study emerges at 72 hours. The ALS SOD1(G93A) profile correlation is only seen with late stage disease.

It might be thought that we are getting further away from the specific pathology, in this case AD, and losing transcriptional information that could be of use in the hunt for a therapy. This is however not the case as can be seen when we search the CMAP with a profile composed of genes whose sense change is conserved across the rodent disease models. Combining the severe AD profile and the four rodent neurodegenerative disease model profiles we get a set of 24 genes whose sense change is conserved. This consists of 10 up regulated and 14 down regulated genes, which can be thought of as a binary signature for neuropathology, where +1 is assigned to up-regulated genes and -1 to down-regulated genes, see Table 5. The CMAP drugs with the highest anti-correlation with this signature are shown in Table 6. Remarkably, there are at least 9 neuroprotective agents in the top 22 hits. In particular, Galantamine, a plant alkaloid, is currently prescribed for early stage AD [45-48], it was originally studied for its acetylcholinesterase inhibitory activity, but it may also act on other targets [49]. The flavones chrysin [50], apigenin [50,51] and luteolin [50,52,53] have been reported to have neuroprotective activity. As have the two kinase inhibitors H-7 (PKA/PKC inhibitor)[54] and GW-8510 (CDK inhibitor)[55]. The β-carboline plant alkaloid harmine has several neuronal actions. It acts to slow down the breakdown of monoamine neurotransmitters through inhibition of monoamine oxidase A [56,57]. Also, it has been shown to specifically inhibit DYRK1A, an enzyme responsible for phosphorylation of tau and thereby may act to slow tau pathology in AD and DS [58,59]. Nomifensine is a dopamine reuptake inhibitor originally prescribed as an anti-depressant [60] that has been shown to reverse dopaminergic neurotoxicity [61-63] and to have beneficial effects in Parkinson's disease [64]. Carbachol is an acetylcholine receptor agonist, but with poor blood brain barrier penetration [65]. The possible application of the other high scoring compounds remains to be determined.

**Table 5:** Multi-species derived nerodegenerative signature.

Gene	signature	A	B	C	D	E
**VSNL1**	-1	-1.20	-1.19	-0.28	-0.28	-0.50
**NEFL**	-1	-1.06	-0.72	-0.23	-0.42	-0.60
**CCK**	-1	-0.91	-1.12	-0.47	-0.23	-0.85
**YWHAH**	-1	-0.88	-0.38	-0.26	-0.17	-0.39
**TUBA4A**	-1	-0.75	-0.80	-0.45	-0.32	-0.23
**YWHAB**	-1	-0.73	-0.39	-0.72	-0.21	-0.40
**NRN1**	-1	-0.67	-0.65	-0.67	-0.31	-0.76
**GUCY1B3**	-1	-0.66	-0.64	-0.30	-0.22	-0.13
**SCN1A**	-1	-0.60	-1.05	-0.62	-0.36	-0.35
**SYP**	-1	-0.54	-0.62	-0.49	-0.26	-0.25
**NDRG3**	-1	-0.52	-0.58	-0.31	-0.17	-0.33
**PPP2R2B**	-1	-0.42	-0.66	-0.51	-0.25	-0.27
**MDH2**	-1	-0.42	-0.36	-0.28	-0.13	-0.12
**DYNC1I1**	-1	-0.39	-1.00	-0.71	-0.35	-0.47
						
**RHOC**	+1	0.30	0.39	1.03	0.73	0.74
**SLC16A1**	+1	0.35	0.36	0.69	0.48	0.20
**MAPKAPK2**	+1	0.40	0.32	0.51	0.37	0.23
**CTSB**	+1	0.43	0.79	0.80	0.86	0.57
**DAB2**	+1	0.47	1.05	0.94	0.58	0.29
**CLU**	+1	0.47	0.54	0.37	0.50	0.22
**TBC1D2B**	+1	0.51	0.32	0.42	0.18	0.76
**ZFP36L1**	+1	0.61	0.59	0.98	0.56	0.48
**SREBF1**	+1	0.63	0.90	0.57	0.36	0.46
**MDFIC**	+1	0.63	0.80	1.43	1.17	0.43

The AD based signature consists of a set of down-regulated (-1) and up-regulated (+1) genes based on the conservation of gene sense change across multiple expression studies of neurodegeneration. The five expression profile fold values are shown with: A, severe AD in human; B, chronic spinal chord contusion in rat; C, chronic spinal chord contusion in mouse; D, SOD1(G93A) mutation late stage in mouse; E, prion disease in mouse.

**Table 6:** Multi-species derived nerodegenerative signature can pick out neuroprotective drugs in CMAP.

COMPOUND	< r >	prob	N
verteporfin	-0.48	0.007	3
lysergol	-0.43	0.021	4
N-acetyl-L-leucine	-0.41	0.003	4
**chrysin**	-0.41	0.004	3
pipenzolate bromide	-0.40	0.033	4
milrinone	-0.40	0.030	3
**apigenin**	-0.39	0.017	4
**carbachol**	-0.38	0.006	4
camptothecin	-0.38	0.002	3
chlorzoxazone	-0.37	0.006	4
amiodarone	-0.35	0.002	5
**luteolin**	-0.35	0.008	4
torasemide	-0.35	0.020	4
pridinol	-0.35	0.004	4
**H-7**	-0.34	0.002	4
nialamide	-0.34	0.047	4
**GW-8510**	-0.34	0.003	4
trifluridine	-0.34	0.017	4
**galantamine**	-0.34	0.010	4
metacycline	-0.32	0.046	4
**harmine**	-0.31	0.016	4
**nomifensine**	-0.31	0.023	5

The connectivity map hits for the neurodegeneration signature are given. The correlation <*r *> is the average over the drug replicates and the significance is measured with the Student's t-test. Drugs with established neuroprotective activity are highlighted in bold.

## Discussion and Conclusions

We have collected transcriptional data from diverse platform architectures corresponding to various species. By processing the data into effective fold profiles, with the expression levels factored by the average level over the experimental series and defined over a non-redundant gene list, we can directly compare transcriptional profiles from arbitrary sources. The fundamental principal underlying the utility of this approach is that biological effects can be compared through the corresponding transcriptional changes. This idea underlies the CMAP initiative for matching drug to phenotype by querying a database of drug induced transcriptional profiles with a profile defining the phenotype. We have extended this methodology to include potentially all available transcriptional data. In its current version SPIED contains transcriptional profiles for 106,101 arrays covering five platform architectures and three species. This can be easily extended to include other platforms and species. The results largely confirm the hypothesis that high scoring correlations correspond to similar biological processes. We have presented SPIED results for drug perturbagen induced profile queries and queries derived from disease states. For brevity we focussed on three sets of drug treatment profiles corresponding to mTOR/PI3K, estrogen and HDAC inhibitors. SPIED searches with these queries showed correlations with other drug treatments belonging to the same classes and in the case of the mTOR antagonist rapamycin we found high anti-correlations with the profile of a cancer inducing fusion transformation, suggesting a novel indication for rapamycin. Also, for brevity of exposition we focussed on two completely unrelated classes of pathology: cancer and neurodegeneration. In the case of leukaemia we show that a corticosteroid resistance signature derived from leukaemia cell cultures shows significant correlation with a lung cancer predisposition profile and a pancreatic cancer profile. Thereby implicating glucocorticoid resistance in these two pathologies. To illustrate the application of SPIED to neurodegenerative pathology we constructed a severe stage AD profile from a published study. Interestingly, querying SPIED resulted in high correlations with other neuropathological conditions indicating a common feature of synaptic loss and mitochondrial dysfunction. Restricting our searches to the rodent subset of SPIED returned expression profiles from animal models of neurodegeneration and neuronal injury. Combining the human and rodent signatures we obtained a core signature that we probed against CMAP for neuroprotective agents. Remarkably, we found at least 9 neuroprotective agents in the top 22 anti-correlating CMAP hits. These results motivate the extension of SPIED and the extension of the CMAP to include other cell types, for example a neuronal cell lineage will be more appropriate for generating drug profiles for neurological diseases. The correlation query scores maybe insensitive to a radical reduction in the number of probes and this should motivate the design of reduced and more cost effective arrays for more extensive data generation.

## Methods

### Compiling the data

Microarray sample files, GSM files, were downloaded form the NCBI GEO database. Individual GSM files were assigned to GSE series and log scaled values scaled to linear and low level responders dropped. EF profiles were then generated based on ratio of individual condition to the average across the series. Expression data from five Affymetrix GeneChip platforms corresponding to three species were collected. These were: all samples from two Human array platforms corresponding to Human Genome U133 Array Set HG-U133A GPL96 (27,337 samples) and U133 Plus 2.0 Array GPL570 (55,196 samples); all samples from the Mouse Genome 430 2.0 Array GPL1261 (21,219 samples) chip; all samples from two Rat chips corresponding to Rat Genome U34 Array GPL85 (2,827 samples) and Rat Genome 230 2.0 Array GPL1355 (7,476 samples). The database thus totals 106,101 samples. Of course, this can always be extended to include more platforms from the same species and/or other species.

### Non-redundant database

The individual GSM sample file expression values were transformed into EF values corresponding to the expression relative to the series mean. Expression values that have been logarithmically transformed are transformed back to a linear scale and low expression values dropped, that is are set to zero and don't contribute to the fold profile. We found that the results were relatively insensitive to the cut-off value and we set this to be 10% of the average expression value. All sample expression profiles within a series were scaled to the same average. The fold vales are defined as$f_{k}=2\frac{s_{k}-\bar{s}}{s_{k}+\bar{s}}$[13], where *s_k _*is the expression level of the *k*^*th *^probe set and $\bar{s}=\frac{1}{n}\overset{n}{\underset{k=1}{\sum }}s_{k}$is the average over the series. For the database to be searchable with cross platform response profiles and gene lists it has to be rewritten as a database of expression profiles over non-redundant gene lists. The EF profiles across the probe sets were therefore mapped onto expression profiles for a non-redundant gene list. In general each gene is represented by multiple probe sets. For each platform we generated the EF statistics for each probe set across the totality of samples. The probe set with the most robust response across the samples was chosen to represent the gene. Explicitly, the probe set with the highest root mean square deviation form zero was chosen to represent the given gene. The number of genes defined on each platform were as follows: GPL96 11,807, GPL570 15,983 genes, GPL1261 13,202 genes, GPL85 chip with 3,844 genes, GPL1355 chip with 6,341 genes. The database totals 106,101 samples and is searchable on a reasonably fast desktop PC in ~10 minutes per query.

### Searching the database

The query profile is a statistically thresholded non-redundant list of genes and associated fold values. Statistical significance is assigned to a fold change based on a simple Student's t-test between multiple control and treatment sample expression values. This is compared to each profile in the database by means of a simple Pearson regression analysis, with a correlation coefficient *r*. The experiments are ranked according to the significance. The significance is measured by scaling the correlation to the normal by a Fisher transformation and measuring the number of standard deviations from the mean. The Fisher transformation is $r^{\prime }=\frac{1}{2}\ln\left(\frac{1+r}{1-r}\right)$ and the standard deviation is$\frac{1}{\sqrt{N-3}}$, where *r *is the Pearson correlation coefficient and *N *is the number of genes making up the correlation. The final ranking score is $s=\frac{\sqrt{N-3}}{2}\left|\ln\left(\frac{1+r}{1-r}\right)\right|$.

### CMAP combined profiles

The CMAP contains ranked lists of probes for 6,100 separate perturbagen treatments of four different human cell lines, with the ranking based on response level relative to control. The treatments are various multiples of 1,306 different drug-like compounds. To generate responder sets that can be used to search SPIED we combined rankings for each separate compound treatment and converted these into pseudo-fold values with associated statistics. The pseudo-fold value is defined by$f_{i}=1-2\frac{r_{i}-\min}{\max-\min}$, where *r_i _*is the rank of the *i^th ^*gene and min/max are the minimal/maximal ranks. Remembering that the highest rank corresponds to the most up-regulated gene. The SPIED was searched with CMAP profiles corresponding to folds with a *p *< 0.05 threshold and with at least three replicates. This left 1,218 separate perturbagen probes. We sought to cluster the perturbagens based on predicted target and response profile similarity. The profiles are given in the additional file 1 file.

### Availability of SPIED

The SPIED database and associated executables are available for download from ftp://ftp.hostedftp.com/~GWftpFILES/SPIED/. The download consists of the SPIED database together with executables for searching SPIED. Source code files to generate the database and perform query searches are provided together with the executables. Documentation on the database, the executables and source code files is also included.

## Authors' contributions

GW is the sole author of the present study.

## Supplementary Material

Additional file 1 **Expression profiles derived from CMAP and published AD studies**. Expression profile signatures derived from pooling CMAP compound treatment replicates, form corticosteroid resistance studies and from various AD stages referenced to age matched controls. These profiles are used to query SPIED. The data is given as excel spreadsheets. Click here for file

Additional file 2 **Top scoring correlations for various expression signature queries against SPIED**. The results upon querying SPIED with expression profiles derived from the signatures in additional file 1. The data is given as excel spreadsheets. Click here for file
